# HIGHER PHYSICAL FITNESS CLUSTERS SHOWED GREATER GLOBAL COGNITIVE OUTCOMES IN MIDDLE- TO OLDER-AGED ADULTS

**DOI:** 10.1093/geroni/igac059.2260

**Published:** 2022-12-20

**Authors:** Joshua Gills, Megan Jones, Anthony Campitelli, Sally Paulson, Kelsey Birk, Jennifer Myers, Jordan Glenn, Michelle Gray

**Affiliations:** University of Arkansas, Fayetteville, Arkansas, United States; University of Arkansas, Fayetteville, Arkansas, United States; University of Arkansas, Fayetteville, Arkansas, United States; St. Elizabeth Healthcare, Cincinnati, Ohio, United States; Neurotrack Technologies, Inc., Redwoods City, California, United States; Neurotrack Technologies, Redwood City, California, United States; Neurotrack Technologies, Redwood City, California, United States; University of Arkansas, Fayetteville, Arkansas, United States

## Abstract

Approximately, 6 million individuals in the United States are living with Alzheimer’s disease. A new diagnosis occurs every 67 seconds, which will triple the rates by 2050. Recently, physical dysfunction has been associated with cognitive decline; however, usually, this is examined in one dimension of physical fitness (PF). A more robust way including multiple domains of PF would be beneficial in examining the relationship between PF and cognition. Therefore, the purpose of this investigation was to examine cognition in clustered PF variables among middle to older-aged adults. Participants (n=216;73% female) enrolled and completed a DXA scan, RBANS, handgrip, sit-to-stand power with TENDO, dual-task (4-meter and 10-meter), and 6-minute walk distance test. A hierarchal cluster analysis was utilized to identify PF cluster for participants, a one-way ANOVA was used to assess differences in cognition between clusters. Cluster 1 (C1;n=29) was characterized with the highest physical fitness values, cluster 2 (C2;n=74) was in-between C1 and C3, cluster 3 (C3;n=113) had the lowest values among PF variables. C1 had significantly higher global cognitive and visuospatial scores compared to C3 (p< 0.05). C1 and C2 had significantly higher values on line orientation and figure recall than C3 (p< 0.05). Data showed high PF clusters had higher global cognitive values when compared to lower PF clusters. Moreover, higher PF showed greater visuospatial and delayed memory values compared to lower PF. Clustering PF tasks served as a practical tool evaluating cognition—this may be useful for future interpretation of cognitive decline where higher PF represents higher overall cognition.

